# The PIN domain endonuclease Utp24 cleaves pre-ribosomal RNA at two coupled sites in yeast and humans

**DOI:** 10.1093/nar/gkw645

**Published:** 2016-07-14

**Authors:** Graeme R. Wells, Franziska Weichmann, David Colvin, Katherine E. Sloan, Grzegorz Kudla, David Tollervey, Nicholas J. Watkins, Claudia Schneider

**Affiliations:** 1 Institute for Cell and Molecular Biosciences, Newcastle University, Newcastle upon Tyne NE2 4HH, UK; 2 Wellcome Trust Centre for Cell Biology, University of Edinburgh, Edinburgh EH9 3JR, UK; 3 MRC Institute of Genetics and Molecular Medicine, University of Edinburgh, Edinburgh EH4 2XU, UK

*Nucl. Acids Res*. (2016) 44 (11): 5399–5409. doi: 10.1093/nar/gkw213

On two occasions (page 5405), the authors have misquoted a residue in Rcl1 that was shown to be important for Bms1 interaction. The correct residue is R327, and not R237 as entered in the article. The correct sentences are shown below.

Recent mutational analysis of Rcl1 based on the crystal structure of the yeast Rcl1-Bms1 dimer showed that mutation of **R327**, within the RDK motif, disrupts interaction with Bms1 (10). The Rcl1-Bms1 interaction is required for nuclear import of Rcl1 (34) and, consistent with this, an **R327A** mutation severely impaired the nucleolar localization of yeast Rcl1 (10).

10. Delprato,A., Al Kadri,Y., Perebaskine,N., Monfoulet,C., Henry,Y., Henras,A.K. and Fribourg,S. (2014) Crucial role of the Rcl1p-Bms1p interaction for yeast pre-ribosomal RNA processing. *Nucleic Acids Res*., **42**, 10161–10172.

34. Karbstein,K., Jonas,S. and Doudna,J.A. (2005) An essential GTPase promotes assembly of preribosomal RNA processing complexes. *Mol. Cell*, **20**, 633–643.

